# Correction to “Safety and Efficacy of REGENDIL Infused Hair Growth Promoting Product in Adult Human Subject Having Hair Fall Complaints (Alopecia)”

**DOI:** 10.1111/jocd.70064

**Published:** 2025-02-24

A. Merja, N. Patel, M. Patel, S. Patnaik, A. Ahmed, and S. Maulekhi, “Safety and Efficacy of REGENDIL Infused Hair Growth Promoting Product in Adult Human Subjects Having Hair Fall Complaints (Alopecia).” *Journal of Cosmetic Dermatology* 23 (2024): 938–948, https://doi.org/10.1111/jocd.16084.

The authors noticed a typographical error in Table 1 on page 940, representing the “Demographic Data of the subjects”. The units for Weight and Height were incorrectly listed as “Weight (cm)” and “Height (kg).” This should be corrected to “Weight (kg)” and “Height (cm).”

The revised version of Table 1 is shown below.

**TABLE 1:** 

Parameter	Statistic	*N* = 29
Gender *n* (%)	Female mean (SD)	15 (51.7%)
	Male mean (SD)	14 (48.3%)
Race *n* (%)	Asian mean (SD)	29 (100%)
Age (years)	Mean (SD)	35.8 (5.70)
Weight (kg)	Mean (SD)	61.7 (13.6)
Height (cm)	Mean (SD)	165 (10.1)

Abbreviation: SD, standard deviation.

We apologize for this error.

